# The compliance of nutrition claims on pita bread in Lebanon and risk on public health: a cross-sectional study

**DOI:** 10.1186/s40795-022-00526-7

**Published:** 2022-04-17

**Authors:** Priscilla Bedran, Christelle Bou-Mitri, Samar Merhi, Jacqueline Doumit, Jessy El Hayek Fares, Antoine G. Farhat

**Affiliations:** 1 Faculty of Nursing and Health Sciences, Notre Dame University-Louaize, Zouk Mosbeh, P.O.Box: 72, Zouk Mikael, Lebanon; 2 Department of Health and Medical Sciences, Khawarizmi International College, Abu Dhabi, United Arab Emirates

**Keywords:** Nutrition claims, Health claims, Bread, Pita bread, Labelling, Sugar, Fiber, Salt, Protein

## Abstract

**Background:**

Mislabeling is a type of fraud, that can lead to major health concerns, especially when used on staple foods like bread. This study aimed to assess the compliance of nutrition claims on pre-packaged Pita bread in Mount Lebanon with national (LIBNOR; NL 661:2017) and international (CODEX; CAC/GL -2–1985) standards.

**Methods:**

A cross-sectional study was conducted and Lebanese bread samples (*n* = 75) were collected from all the registered bakeries in Mount Lebanon directorate (*n* = 25). The claim compliance assessment was based on values of the nutrition facts panel and standard nutrient analyses, following official methods.

**Results:**

Of all assessed breads, 84% carried nutrition claims, and 25.3% carried health claims. Among nutrition claims, 70.7% had non-addition claims, 56.0% had nutrient content claims, and 1.3% had comparative claims. The results showed a high prevalence of nutrition claims with majority non-compliant. Based on the nutrition facts panel, only 32.4% of the sugar related claims, 45.5% of the fiber claims, and 54.4% of salt claims were eligible to make those statements. Based on the chemical nutrient analyses, only 47.0% of sugar claims, 16.1% of fiber claims, and 37.5% of salt claims were compliant. All the claims related to protein (*n* = 7) were compliant.

**Conclusions:**

These results suggest the urgent need to develop clear guidelines for the effective implementation of the current standard; in order to prevent mislead consumers from making poor decisions at the point-of-sale, which might affect their overall health and efforts towards proper nutrition.

## Background

Unhealthy dietary choices, and lifestyles are key risk factors in the development of chronic non-communicable diseases (NCDs), which are increasingly prevalent in low- and middle-income countries, especially in the Mediterranean and North African (MENA) region [1–3].

Nutrition labelling including nutrition and health claims (NHC) is one of the tools used to promote healthy eating, by helping consumers make informed decisions at the point-of-sale [4].

A claim is any statement made on a product suggesting that the latter has any special characteristic regarding its nutritional content, origin, composition or any other quality [5]. Different types of claims can be found on food products, including health claims, and nutrition claims; the latter are divided into nutrient content claims, comparative claims, and non-addition claims [6].

Claims on food labels should be truthful and not misleading [6]. Furthermore, claims should be formulated in a way that allows consumers to understand the beneficial effects of the product [7]. Food labelling regulations had been implemented in various countries to protect consumers from any fraudulent information. Non-compliance of claims with the standards and guidelines have been reported in several developed countries like Canada, Australia, Slovenia, and the United Kingdom [3, 8–11], as well as in developing countries like Honduras, Malawi and Mongolia [12, 13]. Only few studies determined compliance based on nutrient assessment, and similar assessments conducted in MENA countries were modest. For instance, only 38% of nutritional labels were compliant with Saudi regulations [14].

In Lebanon, 6% of gluten-free labelled food products (*n* = 173) were fraudulent [15], whereas 100% of sodium related claims on bread (*n* = 48), were found to be credible [16]. A previous study assessing the NHC on pre-packaged bread, in Lebanon, reported high exposure of consumer to these claims, with 59.6% of the assessed samples (*n* = 354) carrying at least one claim [17]. Most of the nutrition claims were related to sugar, salt and fiber [17].

Bread is a staple food worldwide, and a major vehicle for nutrients such as sodium, sugar and fiber [9, 16, 18]. Knowing that such nutrients are critical, and directly linked to various NCDs, their misuse on staple food could lead to major public health concerns if not controlled properly [19–21].

The Lebanese Standards Institution (LIBNOR) has set guidelines and standards related to the use of NHC [22]. However, in Lebanon like other developing countries, where resources are scarce, food labelling is not considered a priority, and its control is not implemented on regular basis, until significant breaches are reported. Accordingly, the aim of this study was to assess the compliance of nutrition claims on pre-packaged pita bread in Mount Lebanon with national LIBNOR [22] and international CODEX [23] standards, based on the nutrition facts values and nutrients analyses. Given the influence of NHCs in communicating information that may affect consumer’s health, findings of this study will help highlight the importance of implementing regulations in low- and middle- income countries like Lebanon where time and resources needed to adjust and implement policies that support public health and nutrition are limited.

## Methods

### Materials

Boric acid, potassium sulphate, methyl red, sodium hydroxide (NaOH), and sodium standard for the atomic absorption spectroscopy (AAS) were purchased from Steinheim, Germany. Copper sulphate and methylene blue were obtained from Fisher Chemical, India. Sulphuric acid (H_2_SO_4_) (95%) from Analar Normapur, France; acetone and octanol (99.5%) from Alpha Chimie, (France, cod. 120,291). Deionized water was prepared using WaterPro system from Labconco (Kansas City, Missouri).

### Sample collection

In 2018, a total of 25 bakeries producing pita bread in Mount Lebanon were registered at the Ministry of Industry. All the registered bakeries were visited for pita bread collection. The sampling was all inclusive (*n* = 75), and consisted of randomly choosing one item of all types of pita bread pre-packaged products from each bakery at the point-of-sale. In addition, clear pictures of both sides of the packets were taken [9]. The bread samples were kept in airtight sterile plastic bags and stored at -20 °C until further analyses [24]. Bread of different package sizes were considered as one sample. All the information on the packaged bread were recorded, including the brand name, type of bread, type of grain, claims, availability of nutrition facts panel, and the displayed value of different nutrients [3, 9]. The different types of claims included nutrient content, non-addition, comparative, and health claims. Nutrient content claims are used to refer to a certain level of a nutrient for example “low”, “high”, “source”. Nutrient comparative claims compare two or more products using words like “reduced” and “increased”. Non-addition claims infer that an ingredient that is normally present in this food has not been added during production either directly or indirectly [6]. These claims were classified according to CODEX [6] and LIBNOR [22] standards.

#### Nutrient analyses

Pre-packaged pita bread samples carrying sodium related claim (*n* = 24) were tested for sodium content following the reference method from the American Association of Cereal Chemists AACC 40–71 [25]. A total of 31 pita bread samples with claims related to fiber, were analyzed for fiber determination using the Association of Official Analytical Chemists (AOAC) Official Method 950.37 [26]. Kjeldahl principle was used to assess protein content of 7 pita bread samples with protein claims following the AOAC 920.87 official method [27–30]. Pre-packaged pita bread samples carrying a sugar related claim (*n* = 34) were tested using the AOAC 982.14 method [31].

### Statistical analyses

All collected data were coded and analyzed using the IBM’s Statistical Package for Social Sciences (SPSS) version 22 (IBM, Inc, Chicago, IL). Descriptive analyses were carried to assess the prevalence of different types of claims, claims related to different nutrients, availability of nutrition facts, and medians of different nutrient levels. In addition, descriptive analyses were used to assess compliance with claim criteria, as for the eligibility to make claims based on the nutrition facts and the nutrient analysis. Kruskal–Wallis test was used to assess the difference of nutrient levels between different types of bread. A *p*-value < 0.05 was used for statistical significance.

## Results

### Pre-packaged pita bread sample characteristics and nutrient claims

A total of 75 pre-packaged pita breads were identified in all the registered bakeries (*n* = 25) producing this type of bread, in Mount Lebanon (Table 1). samples (88%) were made from wheat flour. Among the pre-packaged pita breads collected, 46.7% (*n* = 35 out of 75) had a nutrition facts panel available on the package. However, out of 63 breads with a claim, 32 (50.8%) did not have a nutrition facts panel displayed on the package.

**Table 1:** Descriptive statistics for pre-packaged pita bread sample (*n* = 75) collected from bakeries (*n* = 25) across Mount Lebanon

Characteristics		n	%
Type of bread	White	22	29.3
	Whole wheat	16	21.3
	Brown	14	18.7
	Bran	13	17.3
	Other	10	13.3
Type of grain	Wheat	66	88.0
	Oat	5	6.7
	Other	4	5.3
Availability of nutrition facts panel	Yes	35	46.7
	No	40	53.3
Having at least one claim	Yes	63	84.0
	No	12	16.0

### Nutrient assessment based on the nutrition facts panel of different types of pre-packaged pita bread (*n* = 35) in Mount Lebanon

Based on the information provided on the nutrition facts panel, the median and range of the main nutrients in different types of pita bread were assessed (Table 2). The highest fiber content was found in unconventional types of bread like quinoa, oat, almond and multi-cereal, with a median of 6.5 g.100 g^−1^. As for the sugar content, it was shown to be the highest in white bread, and the lowest in bran bread (medians = 3.2 g.100 g^−1^ and 0.1 g.100 g^−1^ respectively). The highest sodium content was found in white bread (263.8 mg.100 g^−1^); however, the difference in sodium content between different types of bread was not statistically significant (*p* = 0.908). Furthermore, the protein content was statistically the highest for the unconventional types of bread, followed by bran bread.

**Table 2:** Median and range of nutrients according to the nutrition facts panel of pita bread (*n* = 35)

		Median (Range)				
Bread type	n	Fiber (g.100 g^−1^)	Sugar (g.100 g^−1^)	Sodium (mg.100 g^−1^)	Protein (g.100 g^−1^)	Cholesterol (mg.100 g^−1^)
White	6	2.5 (1.9–4)^c^	3.2 (1.7–4.4)^c^	263.8 (40.0–440.0)	8.5 (6.7–10.0)^e^	0
Whole wheat	9	4.6 (2.4–10.3)	0.3 (0–3.3)^d^	233.0 (0–464.3)	8.4 (6.7–10.0)^c^	0
Brown	5	6.2 (2.5–8.3)^d^	1.6 (0–3.3)	254.4 (80.0–314.2)	8.8 (6.7–10.0)	0
Bran	6	6.1 (2–13.9)^d^	0.1 (0–2)^d^	119.0 (0–479.4)	10.0 (8.5–23.8)^d^	0
Other^a^	9	6.5 (3.1–10.9)^d^	0.7 (0–10.3)	243.9 (47–300.0)	10.3 (7.8–16.7)^d,f^	0
Total	35	4.8 (1.9–13.9)	1 (0–10.31)	249.2 (0–479.4)	9 (6.7–23.8)	0
*p*-value^b^		0.037	0.035	0.908	0.028	0.284

^a^ Other include breads labeled as quinoa, oat, multi-cereal, protein and almond

^b^ Kruskal–Wallis H test (*p* < 0.05)

Non-identical superscripts (c-d/e–f) indicate significantly different bread types

### Prevalence of claims on pre-packaged pita bread in Mount Lebanon (*n* = 75)

Among the collected bread (*n* = 75), 84.0% had at least one nutrition claim, and 25.3% had a health claim (Table 3). The breads also carried non-addition claims (70.7%), nutrient content claims (56.0%) and comparative claims (1.3%). White bread recorded the lowest prevalence of claims (59.1%) as compared to the other types of bread.

**Table 3:** Prevalence of different types of claims on different types of Lebanese pre-packaged pita bread (*n* = 75)

Type of bread	n	Nutrition claims n (%)	Non-addition claims n (%)	Nutrient content claims n (%)	Comparative claims n (%)	Health claims n (%)
White	22	13 (59.1)	12 (54.5)	1 (4.5)	0	0
Whole Wheat	16	15 (93.8)	12 (75.0)	14 (87.5)	0	5 (31.3)
Brown	14	13 (92.9)	9 (64.3)	8 (57.1)	0	3 (21.4)
Bran	13	12 (92.3)	10 (76.9)	9 (69.2)	1 (7.7)	5 (38.5)
Other^a^	10	10 (100.0)	10 (100.0)	10 (100.0)	0	6 (60.0)
Total	75	63 (84.0)	53 (70.7)	42 (56.0)	1 (1.3)	19 (25.3)

^a^ Other include breads labeled as quinoa, oat, multi-cereal, protein and almond

The nutrient content claims were related to sugar (45.3%), fiber (41.3%), salt (32%), cholesterol (16%), protein (9.3%), and gluten (1.3%).

In addition, 62.7% (*n* = 47) of bread displayed a claim related to the absence of preservatives (Table 4).

**Table 4:** Percentage and count of no preservative added claims on different types of pita bread (*n* = 47)

Type of bread	No preservatives added claims	
	n	%
White	12	25.5
Whole wheat	10	21.3
Brown	9	19.1
Bran	9	19.1
Other^a^	7	15
Total	47	100

^a^Other include breads labeled as quinoa, oat, multi-cereal, protein and almond

### Compliance of claims on pre-packaged pita bread in Mount Lebanon with national and international standards

Claims related to salt (*n *= 24), fiber (*n* = 31), sugar (*n* = 34), protein (*n* = 7) and cholesterol (*n* = 12) were assessed for meeting claim criteria based on values of the nutrition facts panel, and nutrient analyses. Cholesterol related claims were only evaluated based on the nutrition facts panel (Table 5).

**Table 5:** Percentage and count of Lebanese pita bread meeting claim criteria for different nutrients

Claim on package	Conditions (not more than)^a^	Compliance based on Nutrition Fact panel		Compliance based on analyses		Median (Range)
		Claim & NF n	Compliant n (%)	n	Compliant n (%)	(g.100 g^−1^)
**Sodium**						
Free	0.005 g.100 g^−1^	2	2 (100)	7	0	0.1 (0–0.3)
Very Low	0.04 g.100 g^−1^	1	1 (100)	1	0	0.1
Low	0.12 g.100 g^−1^	7	3 (42.9)	15	9 (60.0)	0.1 (0–0.5)
No Added^b^	n.d.^c^	1	-	1	-	0.04
Total	-	11	6 (54.5)	24	9 (37.5)	0.1 (0–0.5)
**Fiber**						
Source	3 g.100 g^−1^	4	4 (100.0)	4	2 (50.0)	1.4 (0.8–1.8)
Good source^c^	n.d.^c^	9	0	12	0	1.9 (0.5–3)
High source	6 g.100 g^−1^	9	6 (66.7)	15	3 (20.0)	1.8 (0.3–4.2)
Total	-	22	10 (45.5)	31	5 (16.1)	1.8 (0.3–4.2)
**Protein**						
Source	3 g.100 g^−1^	3	3 (100)	3	3 (100)	13.3 (12.7–13.6)
High source	6 g.100 g^−1^	4	4 (100)	4	4 (100)	17 (12.9–37.2)
Total	-	7	7 (100)	7	7 (100)	13.3 (12.7–37.2)
**Sugar**						
Free	0.5 g.100 g^−1^	12	11 (91.7)	26	16 (61.5)	0.5 (0.5–4.3)
Low^c^	n.d.^c^	1	-	2	-	0.5
Reduced^b^	n.d.^c^	1	-	1	-	2.6
No added^b^	n.d.^c^	5	-	5	-	0.5 (3.4–0.5)
Total	-	19	11 (32.4)	34	16 (47.0)	0.5 (0.5–4.3)

^a^ eligibility conditions based on CODEX (CAC/GL 23–1997) and LIBNOR (NL 661:2017)

^b^ cannot be assessed based on nutrition facts and analyses

^c^ n.d. not defined in CODEX (CAC/GL 23–1997) and LIBNOR (NL 661:2017)

#### Compliance of salt claims on pre-packaged pita bread in Mount Lebanon

Based on the nutrition facts evaluation, all “free” (*n* = 2, 100%), “very low” (*n* = 1, 100%) and 42.9% (*n* = 3 out of 7) of “low” claims referring to salt were compliant with the standards conditions. Based on the nutrient analyses, the only bread with “very low in salt” claim and none of the 7 breads with “salt free” claims were eligible to make the claim on the package. Similarly, 40% of the “low in salt” statements did not meet claim criteria conditions. Only one pre-packaged pita bread claimed the non-addition of salt, which is the absence of any ingredient that could contain sodium salts [6, 22]. No claim criteria conditions are defined by LIBNOR [22] or CODEX [6], to assess the compliance of such statements, however, based on the nutrient analyses, the median sodium content of this bread was 0.04 mg. 100 g^−1^, matching the conditions of a “very low sodium” content.

#### Compliance of fiber claims pre-packaged pita bread in Mount Lebanon

The evaluation of the nutrition facts panel showed that all breads (*n* = 4, 100%), claiming to be a “source of fiber”, and 66.7% (*n* = 6 out of 9) of “high source of fiber” claims, were compliant. In contrast, the results of the nutrient analyses showed that 21.4% of bread with a claim of “source of fiber”, and 50% of the bread with a claim of “high source of fiber” met claim criteria values. In addition, 12 samples had a “good source of fiber”; which is not defined neither in CODEX [6] nor LIBNOR [22] and there are no criteria values that could be used to evaluate the compliance of such claims.

#### Compliance of protein claims pre-packaged pita bread in Mount Lebanon

Assessments using both methods, that is, nutrition facts and protein analysis, showed that all “source of protein” (*n* = 3, 100%), and “high source of protein” (*n* = 4, 100%) claims, met claim criteria conditions. Thus, all references related to proteins were credible.

#### Compliance of sugar claims on pre-packaged pita bread in Mount Lebanon

Based on the nutrition facts evaluation, 91.7% (*n* = 11 of 12) of “sugar free” claims were compliant with the standards, whereas 61.5% (*n* = 16 of 26) of those claims were compliant based on the results of the nutrients analyses. Other sugar related claims including “reduced in sugar” (*n* = 1), “no-added sugar” (*n* = 5), and “low in sugar” (*n* = 2) were all ineligible as they were not defined neither in CODEX [6] nor in LIBNOR [22].

#### Compliance of cholesterol claims on pre-packaged pita bread in Mount Lebanon

Among the collected samples, 16.0% (*n* = 12) claimed the absence of cholesterol in their products. Out of the 12 pita breads claiming to be “cholesterol-free”, 11 (91.7%) exhibited a null value for cholesterol in the nutrition facts panel making them eligible to make the stated claim.

### Standard of identity compliance

Pre-packaged bread samples meeting crude fiber criteria of the “Lebanese bread standard of identity” were assessed (Table 6). Among the analyzed breads, 60.0% of brown bread, and 57.1% of bran bread were compliant to the standard of identity specified in LIBNOR [26].

**Table 6:** Pre-packaged pita bread meeting crude fiber criteria of the Lebanese bread standard of identity

Bread type	n	Conditions (%)^a^	Compliance based on analyses	
			n	Compliant n (%)
Brown	14	≥ 1.5	5	3 (60.0)
Bran	13	≥ 2	7	4 (57.1)

^a^ eligibility conditions based on LIBNOR (NL 240:2010)

## Discussion

This study assessed the prevalence and compliance of nutrition claims on pre-packaged pita bread.

The prevalence of nutrition and health claims found in this study was much higher than what was reported in many countries such as Canada [32], UK [33], Slovenia [34], Serbia [4], Ireland [35], and five more European countries [36]. Moreover, the prevalence was higher than that of a previous study conducted in Lebanon, where among 148 pita bread collected in Mount Lebanon in 2017, 65.5% had at least one claim, 40.5% had a nutrition claim, and 16.9% had a health claim [17]. This showed that the use of nutrition claims has doubled between 2017 and 2018 and the health claims use increased by 1.7 folds. The high variability between the prevalence of claims in these studies could be due to the year when the research was conducted, since the use of these claims and release of pertinent regulations is very recent [37].

Moreover, this study showed a higher prevalence of claims on functional and unconventional bread as compared to white bread, in accordance with the literature [3]. Thus, in pita bread as well as in other food categories, the higher prevalence of claims is found on products made with functional ingredients like whole grains, oat, quinoa and bran. This can be explained by the fact that consumers who usually buy such products are less price sensitive than other consumers, and are more concerned about the healthiness of products [38].

Additionally, among nutrition claims, the observed prevalence of nutrient-content claims was higher than what was reported on pre-packed food products in several countries [35, 39–41]. The increase in the use of claims in Lebanon, as well as the high prevalence of nutrition claims as compared to health claims observed in all other countries might be explained by the lack of governmental surveillance and improper regulations [7, 42]. In addition, manufacturers might be taking advantage of the use of claims as strong marketing tool considering its effect to influence consumers’ choice, leading to an increase in sales [43]. In fact, in Lebanon, consumers are highly influenced by claims at the point-of-sale, especially in the case of bread, where 49.8% of consumers (*n* = 400) rely on claims when buying bread [17].

In line with our results, in Australia, the most common nutrient content claims used on bread were “low in sugar”, followed by “a source of dietary fiber” claim [9]. Similarly, a previous study on pita bread collected between 2017 and 2018 in Mount Lebanon found that the main nutrient claims were mainly related to sugar, salt, and fiber, and that non-addition claims were used on all white bread [17]. In addition, the results of the current study showed that many claims were related to the absence of preservatives which are perceived as an unknown personal and social risk [44] and are believed to be harmful to health [45]. Therefore, bakeries are stressing on the use of “preservative-free” or “no added preservatives” claims to address the increased consumers’ concern [40]. In fact, based on LIBNOR [26], the only additives that are allowed in pita bread are the preservatives sodium propionate, and calcium propionate, both of which have antifungal and antimicrobial properties, with no effect on yeast [46].

In the current study, half of the pita bread samples failed to meet the standard requirements of LIBNOR [47] and CODEX [23] which require displaying nutrition facts panels on all pre-packaged foods. Similarly, failure to meet this requirement was observed in Malawi [12]. Nutrition facts are used by some consumers to make product selections that suit their health conditions, thus, they should be displayed on the package [4]. In addition, carrying a nutrition facts panel will give more credibility to the claim and improve the manufacturer’s ability to compete [48].

Different nutrients were assessed based on the nutrition facts panel. The highest fiber content assigned to the unconventional bread is mainly due to the use of composite flours, and grains like quinoa, oat and wheat bran that are rich in fiber [49–51]. Those breads are recommended for consumers seeking to prevent or mitigate certain conditions like cardiovascular diseases (CVDs), diabetes, obesity, constipation and colon cancer [52, 53].

Furthermore, our results regarding the sugar content are in line with other studies reporting lower sugar content in whole grain loaves compared to other loaf breads [9]. Sugar is used in several types of bread, and high levels may be added to white and brown breads as it enhances fermentation, and the sensory characteristics like flavor and color [9, 54, 55]. Regarding sodium assessment, similarly to our results, higher values of sodium in white bread were reported in Lebanon [16]. Likewise, in Australia, white bread was the highest in sodium content as compared to whole grain and gluten-free [9]. As for the protein content, similarly, other studies reported higher concentrations of protein in whole grain breads compared to white bread [9]. The high protein levels recorded in unconventional breads can be the result of using soy flour which contains up to 45% of protein [56].

In the midst of this high prevalence of nutrition claims, their credibility and compliance with the standards become critical. The results using both, the nutrition facts panels and the nutrient analysis showed low compliance of salt, fiber and sugar claims, in contrast to a high compliance for protein related claims. However, among the minority of compliant claims, the most credible claims were “source of fiber”, “source of protein” and “low in sugar”, which is in line with the literature [9, 13, 57].

In contrast to our results on salt claims, a recent study, analyzing bread samples (*n* = 48) reported a mean sodium content of 127 mg.100 g^−1^ in “low-salt” labelled breads, and the absence of sodium in “zero-salt” labelled breads, which indicates the credibility of those claims [16]. However, studies in Slovenia and Australia reported lower compliance of salt claims [9, 10]. Several studies reported In Lebanon, 38.3% and 7% of shoppers examine the sodium content in products in general [58], and of pita bread respectively [17]. Therefore, such mislabeling can jeopardize people’s health, especially when bread as the main salt contributor in the diet and salt reduction initiatives have been conducted to reduce and prevent NCDs [16, 59].

Similarly, the high prevalence of fraudulent statements related to sugar content might severely affect people with certain medical conditions such as diabetes; especially in Lebanon where the latter ranked fourth among the leading causes of death [60]. Moreover, since bread naturally contains sugar, it is suggested to replace “sugar free” claims by “no-added sugars”. It is practically impossible to differentiate between free and added sugar using chemical analyses. Providing information on intrinsic, and added sugar amounts in the nutrition facts panel, similarly to the approaches followed by the US is recommended.

In Lebanon, fiber claims ranked first among the nutrient content claims people actively look for upon purchasing [17]. However, the high prevalence of fraudulent fiber claims found on bread raises concerns for consumers who are searching to increase their fiber intake due to critical health issues like diverticulosis, constipation or type 2 diabetes [20]. Another impact of this mislabeling is the fact that, bread, with functional ingredients like added fiber, is usually more expensive, accordingly, consumers are paying more for these products.

Protein claims showed a 100% compliance, which can be explained by the proper formulation of those products, that is, the use of high protein components like soy flour, bran, and quinoa as indicated in the ingredients lists.

Among “cholesterol free” claims, 91.7% of claims were accurate. It is important to highlight that the standard recipe adopted by all bakeries in Mount Lebanon relies exclusively on plant based ingredients (flour, salt, yeast, sugar, and water) which are cholesterol free [48, 61]. Thus, although “cholesterol-free” claims on pre-packaged pita bread could be considered credible based on the eligibility criteria, their presence is considered misleading and purely displayed for marketing purposes, as the product will be perceived as a healthier option. In order to be more credible towards consumers, it is suggested to remove “cholesterol-free” claims on pre-packaged pita bread, or replace them by more truthful statements such as “naturally free from cholesterol”.

Like many other low- and middle-income countries, in Lebanon labelling literacy is low. In 2017, Hassan and Dimassi reported that Lebanese shoppers (*n* = 748) had low knowledge on how to read and use food labels. Therefore, food manufacturers can easily take advantage of consumers’ ignorance. Accordingly, proper regulations in such countries are necessary for consumer protection.

Finally, results of Lebanese bread standards of identity showed poor compliance highlighting the need to reassess crude fiber content by bakeries, as their non-compliance with the standard of identity requires changing the name of the product.

This study has potential limitations, as the reported findings are not representative of all claims on food products, as only one food category was assessed. However, bread was assessed as it is a Lebanese staple food that is likely to have a high prevalence of claims. Therefore, issues reported in this study may occur in other food categories as well. In addition, the bread was only collected from bakeries located in Mount Lebanon and did not cover other governorates. However, most of those bakeries have several branches across Lebanon and sell their products in large supermarkets. Thus, it can be assumed, that the results were enough to highlight the misuse of claims, and the need for control and surveillance.

On the other hand, this study is the first audit conducted on staple food in the country and covering multiple nutrients.

## Conclusions

The overall findings show that there is a high prevalence of non-compliant claims on pre-packaged pita bread in Mount Lebanon. Most of the nutrient content claims related to salt, fiber, and sugar did not meet standard criteria.

The high exposure to those inaccurate claims found on a staple food in the Lebanese diet, along with the health drawbacks of excessive intake of some of its major nutrients like salt and sugar highlights the need for more stringent regulations related to the use of NHC. Finally, a legal framework is required to guarantee that nutrient content claims are based on scientific evidence. In the absence of supervision on the compliance of claims, further studies should focus on evaluating the prevalence of claims on food with poor nutritional quality as consumers may perceive them as healthy.

## Data Availability

The datasets used and/or analyzed during the current study are available from the corresponding author on reasonable request.

## Abbreviations

AACC: American Association of Cereal Chemists
AAS: Atomic Absorption Spectroscopy
AOAC: Association of Official Analytical Chemists
CAC: Codex Alimentarius Commission
LIBNOR: Lebanese Standards Institution
MENA: Mediterranean and North African
NCDs: Non-communicable diseases
NHC: Nutrition and Health Claims
SPSS: Statistical Package for Social Sciences
WHO: World Health Organization
